# Detecting Respiratory Pathogens for Diagnosing Lower Respiratory Tract Infections at the Point of Care: Challenges and Opportunities

**DOI:** 10.3390/bios15030129

**Published:** 2025-02-20

**Authors:** Francisco M. Bouzada, Bartomeu Mestre, Andreu Vaquer, Sofía Tejada, Roberto de la Rica

**Affiliations:** 1 Multidisciplinary Sepsis Group, Health Research Institute of the Balearic Islands (IdISBa), 07120 Palma de Mallorca, Spain; franciscomanuel.moyano@ssib.es (F.M.B.); bartomeu.mestrec@ssib.es (B.M.); roberto.delarica@ssib.es (R.d.l.R.); 2 Department of Chemistry, University of the Balearic Islands, 07122 Palma de Mallorca, Spain; 3 (CIBERINFEC)—Centro de Investigación Biomédica en Red de Enfermedades Infecciosas, Instituto de Salud Carlos III, 28029 Madrid, Spain

**Keywords:** pneumonia, biosensor, sputum, bacteria, exacerbation

## Abstract

Lower respiratory tract infections (LRTIs) are a leading cause of mortality worldwide, claiming millions of lives each year and imposing significant healthcare costs. Accurate detection of respiratory pathogens is essential for the effective management of LRTIs. However, this process often relies on sputum analysis, which requires extensive pretreatment steps. The viscous nature and complex composition of sputum present additional challenges, especially in settings where a rapid diagnosis at the point of care is essential. In this review, we describe the main types of LRTI, highlighting different patient care pathway and points of care. We review current methods for liquefying sputum samples and provide an overview of current commercially available diagnostic tools used in hospitals for LRTI detection. Furthermore, we critically review recent advancements in the literature focused on detecting respiratory pathogens and mechanisms of antimicrobial resistance in sputum, including nucleic acid amplification tests, immunoassays and other innovative approaches. Throughout the paper, we highlight challenges and opportunities associated with developing new biosensor technologies tailored for detecting respiratory pathogens in lower respiratory specimens. By shedding light on these pressing issues, we aim to inspire scientific community to create innovative diagnostic tools to address the urgent healthcare burden of lung diseases.

## 1. Introduction

Lower respiratory tract infections (LRTIs) are among the most significant lung diseases, highlighting them as one of the leading causes of death worldwide. LRTIs include influenza, pneumonia, acute bronchitis such as acute exacerbation of chronic obstructive pulmonary disease (COPD), and acute exacerbation of bronchiectasis [1]. They are primarily caused by pathogenic bacteria such as *Streptococcus pneumoniae*, *Haemophilus influenzae*, *Moraxella catarrhalis*, and *Pseudomonas aeruginosa* [2]. Nevertheless, other microorganisms, such as influenza, SARS-CoV-2, respiratory syncytial virus (RSV), fungi, and mycoplasmas, are also recognized as causative agents of LRTIs [2].

LRTIs are a major cause of death globally, affecting all age groups, with 2.8 million deaths recorded worldwide in 2021 [3]. Pneumonia alone is responsible for 14% of deaths among children under 5 years old [4,5]. These infections also contribute significantly to healthcare costs. Total spending across all respiratory conditions in 2016 was $170.8 billion [6]. Hospitals are also impacted by seasonal viral outbreaks, which can overwhelm emergency services. In hospital settings, respiratory pathogens that have evolved diverse mechanisms of antimicrobial resistance often infect immune-suppressed patients. These cases can rapidly progress to fatal sepsis, which is a key driver of intensive care unit (ICU) utilization and further healthcare costs [7].

Accurate pathogen identification is essential for diagnosing LRTIs. Identifying bacterial infections helps clinicians to administer appropriate antibiotics, while detecting antimicrobial-resistant pathogens is crucial for preserving last-resort treatments, thereby prolonging the effectiveness of current antibiotics [8]. However, detecting the pathogen that causes an LRTIs is challenging because the primary sample from the lower respiratory tract is sputum. Although expectoration can be induced through the inhalation of nebulized saline, some patients (particularly children) struggle to produce sputum [9,10,11]. Furthermore, sputum can be contaminated with saliva, which contains microorganisms and biomarkers that are not found in the lower respiratory tract [12]. In patients who require mechanical ventilation, endotracheal or bronchial aspirates can be obtained through the catheter used for intubation. Regardless of the method used to collect them, sputum samples are considered viscoelastic solids or gels and thus must be liquefied prior to analysis [13,14]. Bronchoalveolar lavage (BAL) samples, obtained by washing the airways with saline, may alleviate this issue; however, acquiring these specimens requires bronchoscopy, an invasive procedure [15].

Complicating matters further, the sputum matrix shows an exuberant complexity that changes wildly depending on the patient, particularly in patients with underlying chronic lung diseases. These patients often experience chronic inflammation in the airways, which can confound the diagnosis of a LRTI. For instance, darker sputum color indicates purulence due to the recruitment of neutrophils to the airways, which may be a result of a LRTI [16]. However, patients with chronic lung damage show neutrophilic inflammation without infection, altering the composition of their sputum and making it difficult to differentiate changes in sputum color caused by a LRTI.

Another challenge in diagnosing LRTIs is reducing diagnostic time. This is particularly urgent in cases where timely antibiotic treatment is crucial [17]. Sputum culture remains the primary method used in hospitals to identify the pathogen responsible for a LRTI, but culturing typically takes at least 24 h, often up to 3 days, making it unsuitable for rapid diagnosis of LRTIs. While polymerase chain reaction (PCR) is faster, it is usually not performed at the bedside, leading to delays in results. This impacts antibiotic prescription, as physicians must administer an initial antibiotic regimen without knowing the pathogen responsible for the infection. In this situation, clinical guidelines recommend prescribing empirical antibiotics according to risk factors [18,19]. This may result in overusing last-resort antibiotics in patients who do not need them, exacerbating antimicrobial resistance, or inadequate antibiotic coverage for patients in need, leading to poor outcomes.

**Figure 1:**
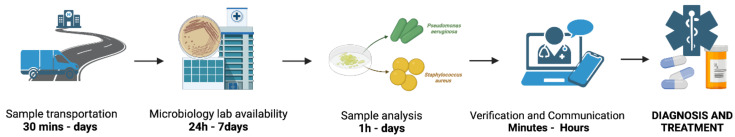
Delays to diagnoses related to performing analyses in centralized laboratories.

Performing analyses at the point of care expedites the diagnosis. Figure 1 highlights potential delays associated with performing analyses in central laboratories. The first delay occurs during sample transportation. Sending samples for analysis can take anywhere from half to an hour, if transported within the same hospital, to several days if sent from another point of care. The second delay involves the availability of microbiology labs. A recent study in Spain found that only 35% of hospitals had microbiology services available 24 h a day, 7 days a week [20]. In other hospitals, if the sample is not processed during the morning shift, analysis is delayed until the next day, and if requested on a Friday, it may not occur until Monday. Time is then required for sample preparation and analysis, followed by verification of results, all of which can also delay the information reaching healthcare providers.

A delayed diagnosis of LRTIs can have serious consequences for patients, particularly those with underlying comorbidities or compromised immune systems [18]. The progression of untreated or improperly treated infections can lead to severe complications such as respiratory failure, septic shock, or multi-organ dysfunction, significantly increasing the risk of mortality [21,22]. Furthermore, delayed treatment can contribute to the development of antimicrobial resistance, as prolonged exposure to suboptimal or inappropriate therapies creates an environment conducive to the survival of resistant pathogens [17,23]. In addition to the clinical risks, delayed diagnosis can also prolong hospital stays, increase healthcare costs, and result in poorer patient outcomes. Therefore, rapid and accurate detection of pathogens at the point of care is crucial to mitigate these risks, ensuring timely and effective treatment that not only improves patient prognosis but also helps combat the growing problem of antimicrobial resistance.

These challenges present an opportunity for developing innovative approaches for LRTI diagnosis. Rapid detection at the point of care is essential for expediting diagnosis and guiding treatment decisions. Fortunately, a new generation of analytical platforms is enabling faster detection of respiratory pathogens in decentralized settings, paving the way for improved LRTI management [24]. In this manuscript, we will review key patient care pathway and points of care in LRTI management, followed by an examination of current specimen preparation and analysis methods. We will then discuss innovative diagnostic approaches, including the use of sensors and biosensors to analyze sputum, and conclude with a general discussion, conclusions, and future perspectives. By shedding light on these pressing issues, we aim to assist experts in analytical chemistry in understanding the complexities of LRTI diagnosis and spur new approaches to solve this urgent issue in current healthcare.

## 2. LRTIs: Diagnostic Approaches According to the Type of Infection

Diagnostic needs for LRTIs are influenced by factors such as geographical location, the setting where the infection was acquired, and the presence of underlying comorbidities [25]. Geographical location matters for various reasons. Some pathogens are more prevalent in certain areas than others. For instance, diagnosing infections caused by *Mycobacterium tuberculosis* is particularly important in low- and middle-income countries, where a third of community-acquired pneumonia (CAP) cases are attributed to this pathogen [26,27]. Additionally, antimicrobial resistance patterns vary by location. In Southern Europe, for example, pathogens are more likely to be resistant to antibiotics compared to other regions of the continent [28,29]. Therefore, strategies for detecting specific resistance mechanisms may be more relevant in some locations. Geographical location also relates to healthcare provision, with some countries allocating more resources to healthcare than others. While an in-depth analysis of these geographical factors is beyond the scope of this manuscript, they are frequently addressed in comprehensive reviews in the literature [30,31].

The diagnostic approach also depends on the setting where the patient was infected [31]. Pneumonia acquired outside healthcare settings is generally referred to as CAP, whereas pneumonia acquired in healthcare environments is called hospital-acquired pneumonia (HAP). Patients requiring invasive mechanical ventilation may develop ventilator-associated pneumonia (VAP). Additionally, patients with chronic respiratory diseases such as COPD and bronchiectasis may experience exacerbations. These are sudden or acute worsening of respiratory symptoms, typically associated with respiratory infections. Exacerbations increase breathing difficulty and may require urgent medical attention. There are significant differences in the patient care pathway for CAP, HAP, VAP, and exacerbations in COPD and bronchiectasis, that should be considered when developing new diagnostic tools [32].

### 2.1. CAP

Emergency department is often the first point of healthcare contact for CAP patients. Despite the geographical disparities, *S. pneumoniae* remains a predominant pathogen globally in all ages [33]. *H. influenzae*, *Staphylococcus aureus*, *M. catarrhalis*, *Klebsiella pneumoniae*, and *P. aeruginosa* are other pathogens contributing to the majority of bacteria CAP aetiology. *Legionella pneumophila* is the most important atypical pathogen for patients with impaired T-lymphocyte function, and it may be either community or hospital acquired. A subset of bacterial pathogens that are resistant to multiple antimicrobial agents (*Pseudomonas*, Enterobacteriaceae, methicillin-resistant *S. aureus*), are of major concern due to challenging antimicrobial therapy [33]. For example, some strains of *S. pneumoniae* are resistant to penicillins, which can affect the choice of antibiotics [34]. Moreover, viruses are the most common cause of CAP, especially in children younger than 5 years [35]. The RSV is the most common viral cause of CAP especially in young children. Other viral pathogens include parainfluenza viruses 1, 2, and 3, influenza A and B viruses, adenovirus, rhinovirus, coronaviruses, and enterovirus.

Several considerations should be kept in mind when designing diagnostic tools for CAP. First, distinguishing between bacterial and viral infections is essential to guide antibiotic treatment decisions. Second, emergency departments are often busy and can become overwhelmed, especially during seasonal viral outbreaks, which are a main cause of pneumonia. Technologies for diagnosing CAP should integrate seamlessly into this workflow, offering rapid results and user-friendly interfaces. Lateral flow immunoassays (LFIAs) are commonly used at this point of health care due to their ease of use, rapidness, and lack of requirement for specialized equipment to interpret results [36].

### 2.2. HAP

The patient care pathway for HAP is different, and these differences must be taken into consideration when developing diagnostic tools for LRTIs. Patients with HAP often have underlying comorbidities, which may lead to immunosuppression either due to conditions like immunodeficiencies, or as a side effect of treatments like those used for cancer. Consequently, HAP patients are more susceptible to bacterial infections that are less common in CAP, such as *S. aureus*, *P. aeruginosa* and *K. pneumoniae* [37,38]. Moreover, bacterial strains in hospitals have evolved mechanisms of antimicrobial resistance due to the frequent use of antibiotics, which can render treatments less effective. Biomarkers used to diagnose infections, or inflammation may also be altered by the underlying comorbidities, making diagnosis more difficult. For instance, cancer patients may have dysregulated body temperature that makes it difficult to identify infection-related fever [39]. Because patients are already hospitalized and under close observation, bedside analysis is often less urgent than it is for CAP patients. Diagnostic technologies for HAP should therefore focus on improving the rapid diagnosis of this potentially life-threatening condition and identifying antimicrobial-resistant pathogens as quickly as possible to inform appropriate antibiotic therapy [37].

### 2.3. VAP

Patients on mechanical ventilation are at an even higher risk of bacterial infection. Intubation provides a direct pathway to obtain endotracheal or bronchial aspirates, which is useful for diagnosis [40]. However, the intubation tube can also provide a surface for bacteria to grow, particularly *P. aeruginosa*, which is highly prevalent in VAP [41]. Interestingly, the presence of bacteria in respiratory secretions does not automatically indicate infection; the bacteria could be transiently colonizing the airways rather than causing an active infection [42]. Differentiating between these two situations is critical to avoid unnecessary overuse of antibiotics. Additionally, most VAP patients are in ICUs, where bacteria are frequently resistant to last-line antibiotics [43]. As a result, diagnostic technologies for VAP must be capable of detecting multiple antimicrobial resistance mechanisms simultaneously, including those related to last-generation antibiotics such as the production of carbapenemases [43]. Furthermore, patients in the ICU have severe comorbidities, which could complicate diagnosis as already mentioned for other forms of HAP [44].

### 2.4. Exacerbations in COPD and Bronchiectasis

Patients with COPD and bronchiectasis have difficulties breathing due to irreversible lung damage. COPD is characterized by chronic bronchitis and emphysema, whereas bronchiectasis involves the dilation of the bronchi. Both conditions are marked by difficulty breathing (dyspnea), with bronchiectasis also involving an excessive production of respiratory secretions [45]. Around 6–10% of the global population suffers from COPD [46], making it one of the top 5 causes of death worldwide [47]. Bronchiectasis has a prevalence rate ranging from 52.3 to 1000 cases per 100,000 people. In both diseases, LRTIs can lead to acute exacerbations, which must be effectively managed to prevent irreversible lung function loss. While most patients acquire LRTIs in the community, those with COPD and bronchiectasis are particularly vulnerable to bacterial infections, which increase as the disease progresses [48,49]. In bronchiectasis, the accumulation of sputum in the bronchi provides an ideal environment for bacteria growth, particularly *P. aeruginosa*, which causes chronic, hard-to-eradicate infections [50]. Additionally, this pathogen has hypermutable strains that, combined with prolonged antibiotic treatment, contribute to the development of antimicrobial resistance [51]. Biofilm formation complicates eradication efforts further [52]. Some patients may develop chronic bronchial infection (CBI), which may require lifelong inhaled antibiotics treatment [53].

The patient care pathway for COPD and bronchiectasis varies depending on factors such as disease severity, the frequency of exacerbations, and whether a CBI is present. Most patients first seek medical attention at the emergency department, though some are enrolled in telemedicine programs and may call their healthcare provider when exacerbations begin [54]. Some of these patients may never visit the hospital, offering opportunities for at-home diagnostic approaches. By contrast, more fragile patients require closer monitoring and may visit specialized clinics even when they are not experiencing exacerbations. Diagnostic tools to evaluate disease progression are essential for managing these patients, with the detection of bacterial pathogens, particularly *P. aeruginosa*, being necessary to guide appropriate antimicrobial therapies.

**Figure 2:**
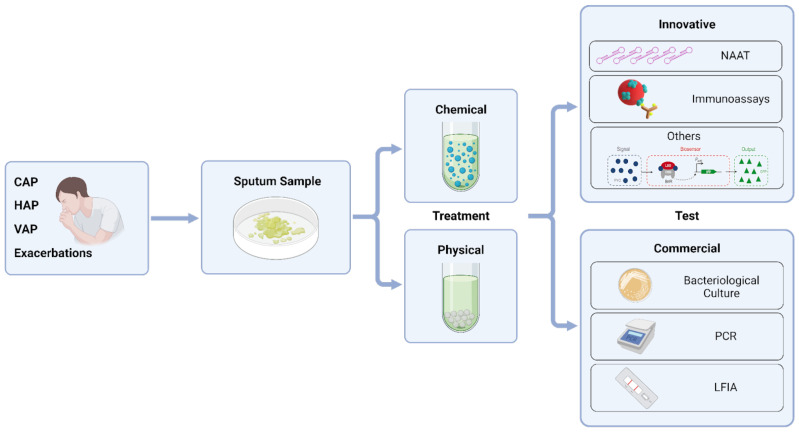
Diagram of sputum analysis for LTRI diagnosis. CAP: Community-acquired Pneumonia; HAP: Hospital-acquired Pneumonia; LFIA: Lateral Flow ImmunoAssay; NAAT: Nucleic Acid Amplification Tests; PCR: Polymerase Chain Reaction; VAP: Ventilator-associated Pneumonia.

In summary, LRTIs are managed in many different healthcare settings, and diagnostic technologies must consider the specific workflows and conditions associated with each setting (Figure 2). The most common causative pathogens vary according to the type of LRTI (CAP, HAP, VAP, and exacerbations), geographical location, and other factors. Developers of diagnostic technologies should factor in regional prevalence of pathogens, as including low-prevalence pathogens in broad diagnostic panels can significantly increase production costs and complicate the validation process required for certification and commercialization. Diagnostic platforms should be designed for point-of-care use, delivering rapid results and seamless integration into existing healthcare workflows. Meanwhile, monitoring tools can be more specialized and remain laboratory based.

## 3. Sputum Processing

Sputum culture remains the primary method used in hospitals to identify the pathogen responsible for a LRTI. It is a viscoelastic solid and therefore needs to be liquefied before analysis. Liquefaction methods can be physical, chemical, or a combination of both. Physical methods usually involve dispersing samples, for example, using glass beads and stirring [55]. They are usually combined with a chemical method and will not be reviewed extensively here. The degree of liquefaction, the time required to complete the process, and the need to implement instrumentation or the potential denaturation of biomolecules are factors that must be considered when choosing a liquefaction method (Table 1).

The mildest sputum treatment approach is lixiviation in a buffer solution [56]. This process extracts a small amount of the target analyte, making it suitable only for subsequent analyses using highly sensitive analytical methods or for analyzing highly abundant targets. Nevertheless, lixiviation is often aided by vortexing or stirring, and the solid component of sputum must be removed by centrifugation. These steps, typically performed in a laboratory setting, make this approach less suitable for point-of-care diagnosis.

Highly cross-linked mucins are the main component of sputum. Traditional liquefaction approaches involve using dithiothreitol (DTT) or N-acetyl cysteine (NAC) to reduce the disulfide bonds cross-linking the proteins [57,58]. This process may be enhanced by heating up the sample, often in combination with vortexing to disperse the specimen. These requirements also make this approach less suitable for point-of-care diagnosis. Although this method is more efficient than simply adding buffers, it typically only achieves partial liquefaction, making quantitative analysis and comparisons between samples or laboratories challenging [59]. Furthermore, reducing agents can denature proteins, which may reduce analytical sensitivity. For example, the sensitivity for detecting IgMs, which contain several domains linked by disulfide bonds, is lower when sputum is liquefied with reducing agents [60].

**Table 1:** Main chemical methods for sputum liquefaction.

Method	Time (min)	Equipment	Denaturing?	References
Buffer	Variable	Centrifuge	No	[53]
DRA	10–30	Vortex and Centrifuge	Yes	[54,56]
Alkaline media	10–30	Vortex and Centrifuge	Yes	[58]
Lytic enzymes	30	Centrifuge	Yes	[55]
Catalase-based	1–2	No	No	[57,59]

DRA: disulfide-reducing agent.

Using alkaline media, usually in combination with a reducing agent, can achieve complete sample liquefaction by denaturing proteins [61]. This method can also be used to inactivate pathogens when this step is required for the diagnosis. However, this harsh treatment can hydrolyze biomolecules, including target analytes or the biorecognition element of a biosensor. Currently, it is mostly commonly used to detect mycobacteria, being also suitable for small analytes like quorum sensing (QS) molecules produced by bacteria, which may not be affected by the alkaline conditions.

Lytic enzymes, such as proteases, are also common in liquefaction reagents, usually in combination with other liquefaction reagents [58]. These enzymes not only help liquefy the specimen but also facilitate cell lysis when detection of intracellular targets is required.

Recently, our group proposed a new liquefaction method for respiratory samples based on oxygen bubble formation using the enzyme catalase [62]. Catalase is naturally present in lower respiratory specimens, being produced by immune cells and some pathogens. The addition of a sufficiently concentrated hydrogen peroxide solution to sputum specimens triggers the generation of oxygen bubbles, which mechanically disperse the specimen. Control experiments with a catalase inhibitor demonstrated that bubble generation is the main mechanism responsible for liquefaction, as no liquefaction occurred in the absence of bubbles. The method does not denature proteins [60], disperses biofilms [62], and does not activate leukocytes [63]. It has been demonstrated that it works well as the pretreatment step of immunoassays, from Enzyme-Linked ImmunoSorbent Assay (ELISA) to flow cytometry and paper-based immunosensors. Furthermore, it is very rapid, achieving liquefaction within 1–2 min, and does not require vortexing or centrifugation, making it an ideal candidate for point-of-care analyses.

In summary, choosing the appropriate liquefaction method is essential for designing new approaches to detect respiratory pathogens. Mild liquefaction approaches, such as lixiviation in buffer, may be used as pretreatment steps when subsequent detection requires live bacteria, provided the detection method is sensitive enough for detecting targets at low concentrations. PCR, which is typically performed in laboratories, can be paired with liquefaction methods that use reducing agents, as these settings usually include the infrastructure required for heating and vortexing. Lastly, immunoassay-based detection methods that require rapid analysis without specialized equipment, such as immunosensors and LFIA, would benefit from the catalase-based liquefaction approach, offering a fast and decentralized solution. Combining these methods or fine-tuning the sample processing step with the addition of components like surfactants, cell-lysing agents, or pathogen inactivators could open new opportunities for more efficient analyses.

## 4. Commercially Available Approaches for Detecting Lower Respiratory Tract Pathogens

Bacteriological culture is the most widely used approach for detecting respiratory bacteria and fungi in hospitals. Samples are usually screened for leukocytes and epithelial cells prior to culture [64]. To this end, specimens are Gram-stained and examined in the microscope. The number of squamous epithelial cells (SEC) and polymorphonuclear leukocytes (PL) are counted. A large number of SEC indicates an excess of saliva and a potential risk of sample contamination from oropharyngeal microbiota. A low infiltration of PL indicates that the sample is not purulent. In both of the cited cases, the specimen is discarded for bacteriological culture. Bacteriological culture in routine analysis is qualitative. The microbiologist counts colonies until the positive threshold is reached (10^5^ CFU mL^−1^ for sputum, 10^4^ CFU mL^−1^ for BAL, and 10^3^ CFU mL^−1^ for bronchial brushing) [65,66]. In the (semi)quantitative approach, colonies are counted in sample dilutions until finding the real number. The precision of this approach, especially for the quantitative culture, is affected by many factors. First, culture only detects alive and metabolically active cells. In other words, cells that do not grow are not detected. For example, cells may not grow as expected if the patient has taken antibiotics prior to sampling the airways. Second, the number of cells that are available for culturing depends on the method used for sample liquefaction. As commented in the previous section, many methods only yield partially liquefied samples. Third, it may be difficult to quantify cells in bacterial biofilms, which do not always grow as colonies. Because of these and other issues, some experts claim that this method is a poor gold standard for respiratory pathogen detection [67,68]. This makes it difficult to evaluate new technologies for respiratory pathogen detection, as comparisons with culture may have limited value.

PCR is routinely used for detecting respiratory viruses in hospitals. Recently, larger PCR panels that not only detect viruses but also bacteria and genes associated with antimicrobial resistance have appeared. For example, the pneumonia panel plus Biofire Filmarray from Biomerieux (Madrid, Spain) can simultaneously detect 34 targets with an assay time within 1 h [69,70]. This approach is more sensitive than culture, having a limit of detection (LOD) in sputum between 10^3^ and 10^4^ cells·mL^−1^ [69]. Several studies have shown that these PCR panels detect more pathogens than bacteriological culture, and it is not clear whether these are false positives or not [71,72]. In some cases, a previous administration of antibiotics may explain why samples are positive when analyzed with PCR and not with culture. In other cases, the lower LOD of PCR may be responsible for the higher frequency of bacterial detection. Furthermore, it is not clear whether these pathogens are colonizing or infecting the airways, making it more difficult to assess whether additional PCR positives are true or false results. Nevertheless, the much shorter turnaround time afforded by PCR has been found to be useful for adjusting antibiotics in some cases [73]. It should also be noted that, despite the astounding multiplexing capabilities of these PCR panels, they still cannot detect all causative pathogens, and therefore there is still a long way for PCR to substitute bacteriological culture.

While LFIA for viruses, performed in nasopharyngeal swabs, are common (especially after the onset of the COVID-19 pandemic), LFIA for sputum samples are rather scarce [74]. The high viscosity and semi-solid nature of sputum may be responsible for this, since it greatly impacts the flow of reagents through the nitrocellulose membrane. Nevertheless, a LFIA for detecting neutrophil elastase is commercialized by the company ProAxsis (Belfast, UK) under the name NEATstik^®^. A study with 124 stable bronchiectasis patients showed that the test results were associated with a significant increase in exacerbation frequency [75]. To the best of our knowledge LFIA for detecting bacteria in sputum are not commercially available. However, LFIAs for detecting *S. pneumoniae* or *L. pneumophila* antigens in urine and Group A Streptococci in throat swabs are commercially available and widely used in hospitals [76,77]. These tests have often been criticized for having modest sensitivity [78]. On one hand, they can only detect one antigen, and serotypes with different antigens are not detected [79]. Some brands have solved this by using a combination of antibodies against different serotypes. On the other hand, a few reports indicate that LFIA are only useful for detecting severe cases due to the low sensitivities reported [80]. Indeed, some researchers have even claimed that using these LFIA may not be cost-effective [81].

LFIAs are also used for the rapid detection of mechanisms of antibiotic resistance, even though the detection is performed after culturing the sample, which can only be performed in microbiology labs. For example, CARBA-5, commercialized by NG biotech, can simultaneously detect 5 carbapenemases, which are enzymes that hydrolyze last resort antibiotics [82]. The analysis requires adding several colonies to a lysis buffer, which releases the enzymes present in the periplasm, followed by analyses with the LFIA. Adaptation of this methodology for direct detection in raw samples would pave the way to performing the assay by the bedside, saving time and improving antibiotic prescriptions from the start.

## 5. Innovative Approaches for Detecting Lower Respiratory Tract Pathogens

In this section, key examples of sputum sensors for pathogen detection will be reviewed. Only approaches tested on real human samples will be considered. Detailed information from the cited examples can be found in Table 2.

### 5.1. Nucleic Acid Amplification Tests (NAAT)

**Figure 3:**
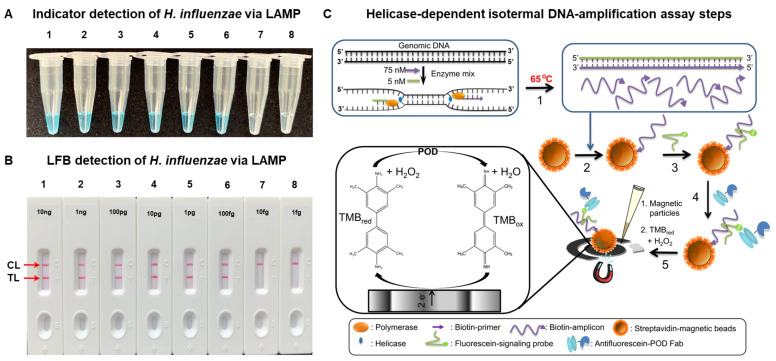
Examples of NAAT for detecting respiratory pathogens in sputum. Sensitivity of the *H. influenzae* LAMP-LFB assay with gradient dilution of genomic DNA templates. Two different methods: (**A**) Colorimetric Indicator; (**B**) Biosensors. Template DNA concentrations for reactions were 10 ng, 1 ng, 100 pg, 10 pg, 1 pg, 100 fg, 10 fg, and 1 fg, respectively. Genomic DNA concentrations from 10 ng to 100 fg produced positive results. CL: Control Line; TL: Test Line. (**C**) Overview of the different steps in the aHDA-electrochemical-genomagnetic assay. LAMP: Loop-Mediated Isotermal Amplification. LFB: Lateral Flow Biosensors. Copyright permission from Springer Nature (**A**,**B**) and from Elsevier (**C**).

A lysis step combined with sputum liquefaction must be performed prior to nucleic acid detection in respiratory samples. Detecting nucleic acids is a powerful tool for pathogen identification, since the sensitivity can be increased by using amplification methods [83], and because of this, bacteriological culture prior to pathogen identification is not needed. Nucleic acid detection also offers the opportunity of identifying pathogens and antimicrobial resistance genes with a single multiplexed analysis [84]. Although detecting antimicrobial resistance genes has the potential to expedite the diagnosis from days to hours, results should be carefully interpreted, since pathogens may not express the detected antimicrobial resistance mechanism, which could lead to the prescription of inadequate antibiotics. For example, the expression of some beta-lactamases is antibiotic-dependent [85], and therefore detecting their gene does not automatically mean that the pathogen has become resistant to the antibiotic they hydrolyze. Traditional nucleic acid amplification methods such as PCR require bulky and expensive equipment for precise temperature control, liquid handling and fluorescent detection. In recent years, researchers have proposed different alternatives to address this issue by simplifying the instrumentation required to perform nucleic acid-based detection. A well-studied approach consists of substituting PCR for isothermal amplification [86], as copies of the target nucleic acid are generated at a fixed temperature. Consequently, a standard heating block, typically available in most laboratories, may be sufficient for temperature control. Excellent review papers exist detailing the different approaches for isothermal nucleic acid amplification [87,88]. For example, a multiple cross displacement amplification (MCDA) method has been proven to be useful for detecting *H. influenzae* by heating at 63 degrees for 40 min using real-time fluorescence detection [89]. The authors achieved a low LOD of 10 CFU per reaction. The authors also compared the results obtained after analyzing 40 DNA samples extracted from sputum using the multiple cross displacement or loop-mediated isothermal amplification (LAMP) and found that MCDA was more sensitive than LAMP (62.5% vs. 57.5%). Yet, the requirement of using real-time fluorescence measurements would make it difficult to implement this approach at the point of care. As an alternative, the use of robust biosensors for detecting the products of isothermal amplification have been also described. Lateral flow biosensors (LFB) are widely used for detecting the products of isothermal amplification reactions. The colorimetric signal transduction mechanism, which relies on the accumulation of gold nanoparticles in test lines, makes this approach easy to implement anywhere, as the results can be interpreted visually. Their implementation for the detection of nucleic acids usually involves performing sample treatment steps with extraction kits followed by an isothermal amplification step, which incorporates labels that are then subsequently detected with the LFB. For example, the detection of *H. influenzae* in sputum with LAMP described in the previous paragraph has been adapted to substitute fluorescent measurements with a LFB, yielding a LOD of 100 fg (Figure 3A) [89]. When tested on 55 clinical sputum samples, the results closely matched those obtained through PCR analysis, thus validating this platform for detecting the target pathogen at lower costs and in decentralized settings (Figure 3B). This alternative has proven to be versatile, as it has been implemented for the detecting *Candida albicans* [90], *P. aeruginosa* [91], *K. pneumoniae* [92], and *M. tuberculosis* [93], among others.

The products of isothermal amplification have also been detected using electrochemical signal transduction mechanisms. This approach has the potential to boost the sensitivity and can be performed with portable readers that are easy to implement at the point of care. *M. tuberculosis* has been detected with electrochemical transducers through hybridization of PCR amplicons of the IS6110 gene to bioreceptors bound to gold electrodes. The sensor showed a LOD of 1.90 nM, with good selectivity for the target pathogen. Yet, the requirement of performing a PCR prior to the detection confines this method to well-equipped laboratory environments. Isothermal amplification of *M. tuberculosis* genes has also been reported using an asymmetric helicase-dependent technique, reaching a LOD of 0.5 aM [94]. The method implemented magnetic particles in order to concentrate the targets and peroxidase as the label (Figure 3C). When tested with real sputum samples, the quantification of nucleic acids with the sensor correlated well with common methods for diagnosing tuberculosis in clinical laboratories.

The integration of lateral flow biosensors (LFB) with multi-cross displacement amplification (MCDA) enabled S. Hu et al. to detect *Acinetobacter baumannii* as well as the carbapenem-resistant gene *bla*_OXA-23-like_. The authors introduced a novel MCDA-based assay, with products analyzed using an LFB that can identify all A. baumannii strains with 100% specificity, a LOD of 100 fg, and a turnaround time of just one hour. This method has been successfully applied to detect the pathogen and its *carbapenemase* in sputum samples [95].

### 5.2. Immunoassays

Detecting pathogens using antibodies as the recognition element is less temperature-sensitive than nucleic acid amplification, although this approach does not benefit from the signal enhancement provided by the latter. Detecting antigens with monoclonal antibodies can be problematic because the composition of antigens may change when the pathogen mutates. This is particularly worrying for pathogens that generate biofilms because the antigenic signature changes drastically with the mucoid phenotype [96]. Furthermore, the target antigen must be available for immunodetection. For example, it would be difficult to detect intracellular targets unless a lysis step is implemented, and surface antigens of cell walls or cell membranes may not be fully accessible for immunorecognition, which can reduce the sensitivity and specificity of the diagnosis.

**Figure 4:**
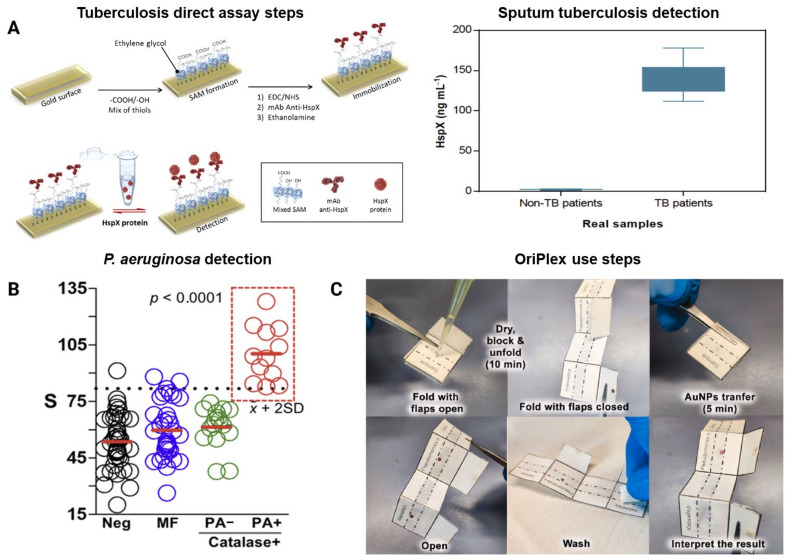
Examples of Immunoassays for detecting respiratory pathogens in sputum. (**A**) Schematic representation of the direct assay used for detecting the HspX protein in pretreated sputum samples. HspX protein concentration levels were analyzed in samples from tuberculosis (TB) patients (n = 12) and non-tuberculosis patients (n = 22). Median, maximum, and minimum values are displayed. (**B**) Detection of *Pseudomonas aeruginosa* using mobile immunosensors in a panel of patient samples. PA+ samples (red) contain *P. aeruginosa* (>10⁵ cells·mL^−**1**^), PA− samples (green) contain catalase-positive bacteria different from *P. aeruginosa* (>10⁵ cells·mL^−**1**^), and MF samples (blue) represent mixed flora. Negative samples (black) were determined by Gram’s stain screening test. Horizontal bars indicate the mean values, with the dotted line denoting signals above two standard deviations from negative samples. Statistical significance was assessed using the Kruskal–Wallis test. (**C**) Step-by-step workflow for bacterial detection using OriPlex. Copyright permission from ACS publications (**A**,**B**) and from Elsevier (**C**).

The Heat shock protein X (HspX) of *M. tuberculosis* has been detected with an immunoassay using a surface plasmon resonance (SPR) platform showing a LOD of 0.63 ng·mL^−1^ and a Limit of Quantification (LOQ) of 2.12 ng·mL^−1^ (Figure 4A) [97].The manuscript described a method for sample treatment and dilution that was required to reduce matrix effects originated by sputum samples. The protocol consisted of adding N-acetyl-L-cysteine, sodium citrate and NaOH to the sample until complete dilution followed by neutralizing with PBS buffer pH 6.8 and centrifugation at 4 degrees for 15 min, using the resulting sediment for analysis. Bacteria were then lysed with 7 pulses of 5.0 m·s^−1^ during 60 s, in a fastprep-24. The supernatants obtained after centrifugation were analyzed. This sample pretreatment approach allowed the authors to accurately classify 12 pre-treated sputum samples from five patients diagnosed with TB, as well as 22 pre-treated sputum samples from non-tuberculous patients, achieving 100% sensitivity and specificity. The results demonstrated an excellent correlation with other laboratory-based analysis methods.

Immunoassays have also been used for detecting *P. aeruginosa* in sputum samples with high sensitivity and specificity in a collection of 104 human sputum specimens (Figure 4B) [62]. In this approach, nanoparticles coated with polyclonal antibodies against the pathogen were stored in paper-based reservoirs. Contact with a paper substrate containing a drop of dried liquefied sputum released the nanoparticles, which were able to identify the bacterial cells after 5 min incubation. Notably, sample liquefaction with the catalase-based approach only required adding the reagent to the sputum sample. No additional centrifugation, vortexing, or lysis step was performed. These features, along with the colorimetric signal transduction mechanism that enabled signal quantification with a smartphone app, make this approach useful for point-of-care diagnosis. Of note, in a follow-up work the simultaneous detection of several respiratory pathogens could be achieved by taking advantage of the folding capabilities of paper (Figure 4C) [98]. This enabled manufacturing an origami biosensor for the simultaneous detection of *P. aeruginosa* and *K. pneumoniae* with LODs of 3.4·10^3^ CFU·mL^−1^ and 1.4·10^2^ CFU·mL^−1^, respectively. The pathogens were also detected after spiking them into bronchial aspirate samples with a concentration of 10^5^ cells·mL^−1^ or higher, thus proving the suitability of this approach for diagnosing LRTIs.

An additional challenge in bacteria detection is identifying the antimicrobial resistance patterns of infectious pathogens. To address this issue, D. Gunasekaran et al. developed an integrated electrochemical chip capable of detecting pathogenic *E. coli* and identifying the presence of β-lactamases. The chip utilizes a specific monoclonal antibody targeting the EspB virulence marker, along with nitrocefin, a substrate specific to β-lactamase. EspB antigen detection is performed using electrochemical impedance spectroscopy, while β-lactam resistance profiling is achieved through voltammetric measurements. Notably, the chip demonstrated a limit of detection of 4.3 ng·mL^−1^ for EspB and 3.6 ng·mL^−1^ for β-lactamases [99].

The QS is a process of communication between cells used by bacteria. The molecules used in QS can be highly specific to the target pathogen, making them excellent targets for bacterial detection [100,101]. Pyocyanin (PYO) is a QS molecule synthesized only by *P. aeruginosa* [102,103,104,105]. The immunoassay format available for detecting PYO is a competitive immunoassay, since PYO is a small molecule with a single epitope. This can make it difficult to detect PYO in complex matrices such as sputum, because negative controls cannot be implemented in competitive immunoassays. For example, a nanoparticle-based immunoassay on paper was able to detect PYO with an assay time below 10 min after liquefying sputum with the catalase-based method [106]. Yet, detecting PYO in spiked samples required diluting them to clearly differentiate spiked specimens from non-spiked ones. It was determined that the matrix not only promoted non-specific interactions with the substrate but also blocked antibody-antigen interactions. Interestingly, performing the immunoassay in paper yielded better results than conventional microtiter plates, thus indicating that non-specific interactions may be substrate dependent.

**Table 2:** Main innovative approaches for pathogen detection in sputum samples.

Pathogen	Target	Sample Treatment	Sample Treatment Time	Assay Type	Transduction Mechanism	LOD	Dynamic Range	Reference
*Haemophilus influenzae*	Gene	NS	NS	LAMP-LFIA	Colorimetric	100 fg	10 ng–1 fg	[86]
*Candida albicans*	Gene	Genomic DNA extraction	40 min	LAMP-LFB	Colorimetric	1 fg	10 ng·μL^−1^–100 ag·μL^−1^	[57]
*Pseudomonas aeruginosa*	Gene	NS	NS	MCDA-LFB	Colorimetric	10 fg	10 ng·μL^−1^–10 fg·μL^−1^	[88]
*Klebsiella pneumoniae*	Gene	NS	NS	MCDA-LFB	Colorimetric	100 fg	10 ng·μL^−1^–0.1 fg·μL^−1^	[89]
*Mycobacterium tuberculosis*	Gene (*IS6110* and *mpb64*)	Alkaline Media	10 min	mLAMP-LFIA	Colorimetric	100 fg	1 ng–100 fg	[90]
*Mycobacterium tuberculosis*	Gene	DRA and Alkaline Media	15 min	LAMP-Electrochemical Detection	Electrochemical	0.5 pM	5–200 pM	[91]
*Pseudomonas aeruginosa*	QS molecule	NS	NS	Immunoassay (Bioluminescent assay)	Fluorescent	~100 mM	NS	[92]
*Mycobacterium tuberculosis*	HspX Antigen	DRA and Alkaline Media	15 min	Immunoassay (SPR)	SPR	0.63 ng·mL^−1^	2–125 ng·mL^−1^	[93]
*Pseudomonas aeruginosa*	Antigen	Catalase-based	1 min	Immunoassay (Origami Immunosensor)	Colorimetric	10^5^ cells·mL^−1^	10^4^–10^9^ cells·mL^−1^	[59]
*Pseudomonas aeruginosa* *Klebsiella pneumoniae*	Antigen	Catalase-based	3 min	Immunoassay (OriPlex Immunosensor)	Colorimetric	3.4·10^3^ CFU·mL^−1^ 1.4·10^2^ CFU·mL^−1^	10^3^–10^8^ CFU·mL^−1^	[94]
*Pseudomonas aeruginosa*	PYO	NS	NS	Immunoassay (ELISA)	Colorimetric	0.07 nM	0.18–2.18 nM	[98]
*Pseudomonas aeruginosa*	PYO	Catalase-based	1 min	Immunoassay (Paper-based Biosensor)	Colorimetric	4.7·10^−3^ µM	4.7·10^−1^–47.6 µM	[101]
*Pseudomonas aeruginosa*	PYO	NS	NS	Fluorometric Biosensor	Fluorimetric	1.3 μM	NS	[102]
*Pseudomonas aeruginosa*	QS molecule (3-oxo-C12-HSL)	Buffer	7 min	Cell-Free Biosensor	Fluorimetric	1.56 nM	5–100 nM	[103]
*Pseudomonas aeruginosa* *Klebsiella pneumoniae* *Enterobacter cloacae* *Citrobacter freundii*	Β- Lactamase	DRA	NS	Plasmonic Nanosensors	SPR	10^−5^ cells·mL^−1^	10^5^–10^−7^ cells⋅mL^−1^	[104]
Bacteria pneumonia Gram-positive Gram-negative	Bacterial Cell Wall	NS	NS	Electrochemical Biosensor	Electrochemical	3.1 CFU·mL^−1^ 3.0 CFU·mL^−1^	10^1^–10^5^ CFU·mL^−1^	[105]

DRA: Disulfide-reducing Agent; ELISA: Enzyme-Linked ImmunoSorbent Assay; HspX: Heat shock protein X; LAMP-LFB: Loop-mediated isothermal Amplification combined with a label-based Lateral Flow biosensor; LOD: Limit of Detection; MCDA-LFB: Multiple Cross Displacement Amplification combined with a label-based Lateral Flow biosensor; mLAMP-LFIA: multiplex Loop-mediated isothermal Amplification combined with a label-based Lateral Flow Immuno-Assay biosensor; NS: Not State; PYO: Pyocyanin; QS: Quorum Sensing; SPR: Surface Plasmon Resonance.

Despite the improvements and various approaches developed in the literature for rapid diagnostics, challenges still remain for the scientific community to address. For a truly rapid and point-of-care diagnostic tool, a user-friendly platform is essential. To achieve this, the manipulation required for the end-user to perform the test must be minimal, which also helps reduce inter-user variability. Additionally, the development of innovative strategies to enhance signal detection and reach the low limits of detection afforded by nucleic acid amplification tests, such as PCR, is crucial. Finally, the robustness of the biosensors, ensuring rapid, selective, and specific detection, is vital. Platforms presented in the literature must not only be tested in controlled environments but also in a variety of real-world scenarios to guarantee consistent performance in all situations.

### 5.3. Detection Based on Other Signal Generation Mechanisms

PYO has a characteristic redox signature, which some authors have exploited in order to detect it with electrochemical transducers. For example, PYO and other redox metabolites from *P. aeruginosa* (2-heptyl-3-hydroxy-4-quinolone (PQS), 2-heptyl-4-hydroxyquinoline (HHQ)) have been electrochemically detected with boron-doped diamond electrodes in the concentration range between 1 and 100 μM. Recoveries of 32%, 43%, and 58% for PYO, HHQ, and PQS, respectively were obtained after analyzing a real sputum sample with known amounts of the three targets, using chloroform to extract the molecules.

**Figure 5:**
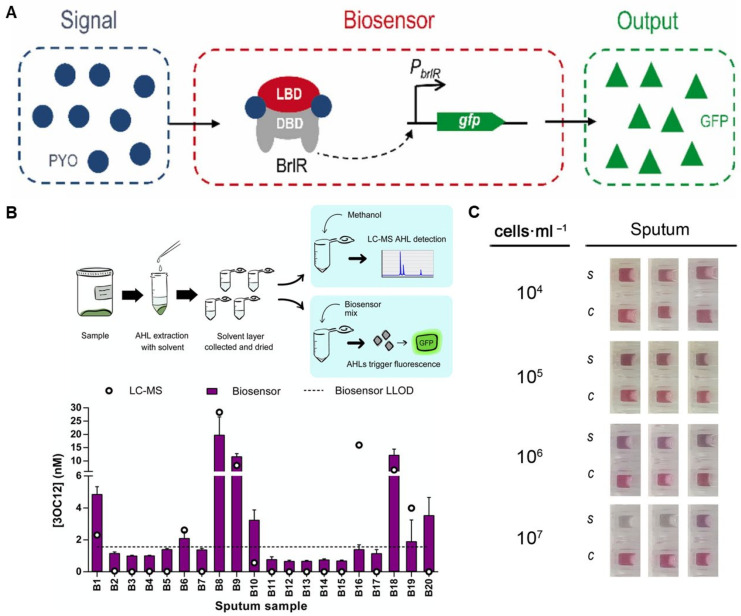
Examples of alternative approaches for detecting respiratory pathogens in sputum. (**A**) PYO sensor-brlR. DBD: DNA-binding domain; LBD: ligand-binding domain; GFP: Green Fluorescent Protein. (**B**) Investigation of AHLs in sputum samples. Workflow for solvent extraction to concentrate AHLs from sputum, followed by concurrent analysis using a cell-free biosensor and LC–MS/MS. Optimization of the cell-free LasRV biosensor for sputum sample analysis. Analysis of 20 CF sputum samples using LasRV in Rosetta cell-free and LC–MS/MS. Biosensor fluorescence output between 30 and 300 min was converted to 3OC12-HSL concentration using a calibration curve. The mean and standard deviation (SD) of n = 3 biosensor reactions per sample are shown. (**C**) Detection of carbapenemase producing bacteria in patient samples. KPC+ E. cloacae spiked at different concentrations into sputum. (i) Photographs of test results (S: samples; C: control). Copyright permission from Elsevier (**A**,**C**) and from ACS publications (**B**).

Recently, a new approach was introduced for detecting PYO that may solve the issue of non-specific interactions with antibodies. It consisted of using whole cells as bioreceptors and signal generators. The cells express a transcriptional regulator (BrlR) responding to PYO by producing green fluorescent protein (GFP) (Figure 5A) [107]. This generated a fluorescent signal that depended on the concentration of PYO with a LOD of 1.3 μM. Good recoveries were observed in real sputum samples. However, this approach is less well suited for diagnoses at the point of care, as it requires several pieces of large equipment that can only be found in central laboratories. This issue could be alleviated by using a cell-free expression system (Figure 5B) [108]. It was shown that a system responsive to the QS molecule 3-oxo-C12-HSL from *P. aeruginosa* could yield a dose-dependent response in the concentration range between 0.1 and 10^4^ nM. When applied to sputum samples from cystic fibrosis patients the biosensors showed good correlation with analyses performed with liquid chromatography coupled to mass spectrometry (LC-MS/MS) after using an extraction method based on adding acidified ethyl acetate or dichloromethane followed by centrifugation. The authors also pointed out that transition to clinical diagnoses could be facilitated in the future by using freeze-dried reagents, on their own or embedded in paper discs.

The production of beta-lactamases is one of the most worrying mechanisms of antimicrobial resistance, since these biocatalysts are able to hydrolyze wide-spectrum antibiotics. Among these, enzymes capable of inactivating carbapenems (i.e., carbapenemases) are particularly worrying in the context of HAP and VAP management, as these antibiotics are often used as the last line of defense against nosocomial pathogens. A colorimetric sensor was proposed to expedite the detection of carbapenemase-producing pathogens at the point of care [108]. It consisted of trapping bacterial cells with positively charged magnetic beads followed by addition of a carbapenem. The hydrolysis of the antibiotic resulted in the generation of acids that lower the pH of the solution. In turn, this could be detected by controlling the state of aggregation of gold nanoparticles in the presence of proteins, which is pH dependent. When applied to liquefied sputum samples, this approach yielded mauve to gray-colored nanoparticles solution when the concentration of carbapenemase-producing pathogen was higher than 10^5^ CFU·mL^−1^, whereas controls were always red-colored (Figure 5C) [109]. The whole assay could be performed within 3 h, which is much faster than culturing cells and obtaining a full antibiogram, which may take several days.

Electrochemical biosensors have also been proposed for detecting antibiotic-resistant bacteria [110]. In this approach, impedimetric transducers were coated with dot polymers and antibiotics that selectively recognize Gram-positive (colistin) or Gram-negative (vancomycin) bacteria. Authors showed that the biosensors could detect Gram-negative bacteria with a LOD of 3.0 CFU·mL^−1^), whereas Gram-positive yielded a LOD of 3.1 CFU·mL^−1^. The biosensors were validated with a collection of endotracheal aspirate and sputum samples treated with saline. To detect antibiotic-resistant bacteria, authors incubated the biosensors and bacteria with antibiotics at 37 degrees for 30 min. Less pronounced changes in resistance were observed, and authors attributed this change in signal to bacterial death in the presence of the antibiotic.

## 6. Discussion

There is an increasing trend towards detecting pathogens in sputum using colorimetric biosensors, with a preference for paper-based assay formats using lateral flow, origami, or other innovative designs [89,90,91,98,106]. This trend is understandable, as colorimetric signal generation mechanisms require minimal instrumentation for signal reading; in some cases, signals can be evaluated with the naked eye [89,90,91]. Alternatively, they can be quantified using smartphones, an accessible technology at most points of care [62]. However, the viscosity of sputum and its impact on fluid flow must be carefully considered when designing LFB.

Noteworthily, approaches that rely on the detection of nucleic acid amplification products require several purification steps prior to analysis with LFBs. While this approach mitigates fluid viscosity issues, it confines these analyses to well-equipped laboratories capable of performing labor-intensive target extraction protocols. Interestingly, these methods may exhibit reduced sensitivity to non-specific interactions between the sputum matrix and three-dimensional nitrocellulose or paper substrate compared to surface-based designs. This was demonstrated for the detection of PYO when comparing paper-based immunoassays with traditional microtiter plate-based ones. Similarly, extremely low concentrations of tuberculosis antigens were detected using magnetic particles as substrates and electrochemical transducers [94]. Although direct comparative data with surface-based detection is limited, trends in the reviewed literature suggest that such approaches offer potential advantages in robustness for point of care diagnostics.

Detecting QS molecules or other bacterial metabolic products secreted extracellularly does not require lysis steps, rendering these methods better suited for decentralized testing. However, the production of these molecules may vary depending on factors such as whether the pathogen is actively infecting or transiently colonizing the airways, biofilm formation, and the presence of other pathogens or even the bacterial strain. While this variability opens new opportunities to explore bacterial ecology, it also complicates the establishment of diagnostic threshold values for identifying new infections. Additionally, many detection methods involve preliminary extraction steps using organic solvents [108], which are challenging to implement consistently at the point of care.

A potential solution to this issue is the detection of bacterial antigens in samples liquefied with the catalase-based method, which currently offers the shortest sample-to-result time [62,98,106]. However this approach involves adding hydrogen peroxide, which is incompatible with analyses that use peroxidase as the label. Similarly, liquefaction methods using DTT or NAC may interfere with signal generation mechanisms reliant on metalloenzymes because thiols will chelate ions that are required for enzyme activity.

In recent years, numerous reviews have explored various aspects of biosensor-based diagnostics, each contributing unique insights. However, many of these works have limitations in scope and clinical applicability, particularly in respiratory infections.

For instance, Fang et al. [111] focused on biosensor-based fungal diagnostic tests for invasive fungal infections but restricting the detection to a certain type of pathogen. Kumar et al. [112] provided a detailed comparison of nanomaterials in electrochemical, optical, and other biosensors but lacked an in-depth discussion of clinical samples. Similarly, Sivakumar et al. and Tarim et al. [113,114] highlighted advancements in optical and electrochemical biosensors [113] but restricted their analysis to airborne pathogen detection excluding clinical respiratory samples, and microfluidic-based methods [114] using biological fluids but only for virus detection in respiratory diseases. Both cases have limitations in focusing on a certain type of biosensors.

In contrast, Gopal et al. [115] discussed biosensors in medical applications, including clinical samples, but focused on rapid diagnostics for antibiotic therapy rather than detecting pathogens for diagnosing respiratory infections. Similarly, Qureshi et al. [116] highlighted nanotechnology-based biosensors for host biomarkers detection, emphasizing sensitivity and selectivity but without a specific focus on pathogen detection only on differentiation of the origin of the infection.

In summary, although these reviews have made valuable contributions to the advancement of biosensor technology, they often concentrate on specific pathogens, technologies, or non-clinical samples. In contrast, our review emphasizes the inclusion of clinical respiratory samples, such as sputum, providing a more direct and relevant approach to diagnosing respiratory infections. This focus bridges a crucial gap in the existing literature and lays a solid foundation for future research in respiratory disease diagnostics using biosensors.

Lastly, while industry efforts are increasingly focused on developing large diagnostic panels, innovative research primarily targets single pathogens or a limited number [98]. The majority of proposed approaches have discrete multiplexing capabilities, with the notable exception of SPR. Consequently, simultaneous detection of multiple pathogens at the point of care remains a significant challenge for LRTI diagnosis.

## 7. Conclusions and Future Directions

In conclusion, while novel approaches in sputum analysis are appearing in the literature, much work is still needed to meet the strict requirements of LRTI diagnosis at the point of care. Many sample pretreatment steps, such as vortexing, cell lysis, nucleic acid isolation, or extraction with organic solvents, are cumbersome, delay the diagnosis, and are difficult to implement at the bedside. New methods employing enzymatic sample liquefaction may provide a solution, as long as they are compatible with the chosen signal generation mechanism. Combining fluidics or designing compact instruments to automate current sample preparation steps may also offer a promising path forward.

Colorimetric signal generation mechanisms currently dominate the field, with numerous examples coupling NAAT with LFB. This approach is advantageous due to the ease of mass production of LFB, and the familiarity of end-users with such devices, facilitating translation from research to clinical applications. However, detecting multiple targets simultaneously remains a challenge with this method. Multitests formats that allow parallel assays, as used for detecting drugs of abuse, could potentially address this limitation. Additionally, examples of SPR and electrochemical biosensors are emerging in the literature. These systems hold promise for advancing the field, particularly as their signal generation mechanisms may be more seamlessly integrated with traditional fluidics for developing fully automated total analysis systems.

Notably, few methods suitable for point-of-care sputum analysis currently address the detection of antimicrobial resistance, an issue of growing importance as therapeutic options for respiratory infections become increasingly limited. Addressing this critical challenge will require collaborative efforts from multidisciplinary teams of clinicians, microbiologists, and technology developers, all focused on advancing respiratory tools at the point of care.

To address the current challenges in LRTI diagnosis at the point of care, further research is essential to focus on the automation of sample preparation steps, particularly in primary care settings where time is a critical factor. The integration of rapid, cost-effective diagnostic technologies, such as electrochemical biosensors and SPR, could facilitate the detection of multiple pathogens and antimicrobial resistance in sputum samples. Furthermore, new collaborations between academic institutions, technology companies, and health organizations should be established to develop point-of-care diagnostic platforms that are scalable and meet regulatory requirements. Continuous training of healthcare staff is also essential to ensure the proper use of these tools, which will improve the effectiveness of diagnosis and treatment of respiratory infections.

## Abbreviations

The following abbreviations are used in this manuscript.

BAL	Bronchoalveolar Lavage
CAP	Community-acquired Pneumonia
CBI	Chronic Bronchial Infection
COPD	Chronic Obstructive Pulmonary Disease
DRA	Disulfide-reducing Agent
DTT	Dithiothreitol
GFP	Green Fluorescent Protein
HAP	Hospital-acquired Pneumonia
HQQ	2-heptyl-4-hydroxyquinoline
HspX	Heat Shock Protein X
ICU	Intensive Care Unit
LAMP	Loop-mediated Isothermal Amplification
LAMP-LFB	Loop-mediated isothermal Amplification combined with a label-based Lateral Flow Biosensor
LC-MS/MS	Liquid Chromatography coupled to Mass Spectrometry
LFB	Lateral Flow Biosensors
LFIAs	Lateral Flow Immunoassays
LOD	Limit of Detection
LOQ	Limit of Quantification
LRTIs	Lower Respiratory Tract Infections
MCDA	Multiple Cross Displacement Amplification
MCDA-LFB	Multiple Cross Displacement Amplification combined with a label-based Lateral Flow Biosensor
mLAMP-LFIA	Multiplex Loop-mediated Isothermal Amplification combined with a label-based Lateral Flow Immuno-Assay biosensor
NAAT	Nucleic Acid Amplification Tests
NAC	N-acetyl Cysteine
NS	Not State
PCR	Polymerase Chain Reaction
PL	Polymorphonuclear Leukocytes
PQS	2-heptyl-3-hydroxy-4-quinolone
PYO	Pyocyanin
QS	Quorum Sensing
RSV	Respiratory Syncytial Virus
SEC	Squamous Epithelial Cells
SPR	Surface Plasmon Resonance
VAP	Ventilator-associated Pneumonia

## References

[1] Feldman C., Shaddock E. (2019). Epidemiology of Lower Respiratory Tract Infections in Adults. Expert Rev. Respir. Med..

[2] Cavallazzi R., Ramirez J.A. (2022). How and When to Manage Respiratory Infections out of Hospital. Eur. Respir. Rev..

[3] GBD 2021 Lower Respiratory Infections and Antimicrobial Resistance Collaborators (2024). Global, Regional, and National Incidence and Mortality Burden of Non-COVID-19 Lower Respiratory Infections and Aetiologies, 1990–2021: A Systematic Analysis from the Global Burden of Disease Study 2021. Lancet Infect. Dis..

[4] Li Y., Wang X., Blau D.M., Caballero M.T., Feikin D.R., Gill C.J., Madhi S.A., Omer S.B., Simões E.A.F., Campbell H. (2022). Global, Regional, and National Disease Burden Estimates of Acute Lower Respiratory Infections Due to Respiratory Syncytial Virus in Children Younger than 5 Years in 2019: A Systematic Analysis. Lancet.

[5] World Health Organization (WHO) Pneumonia in Children. https://www.who.int/news-room/fact-sheets/detail/pneumonia.

[6] Duan K.I., Birger M., Au D.H., Spece L.J., Feemster L.C., Dieleman J.L. (2023). Health Care Spending on Respiratory Diseases in the United States, 1996–2016. Am. J. Respir. Crit. Care Med..

[7] Kumar N.R., Balraj T.A., Kempegowda S.N., Prashant A. (2024). Multidrug-Resistant Sepsis: A Critical Healthcare Challenge. Antibiotics.

[8] Yamin D., Uskoković V., Wakil A.M., Goni M.D., Shamsuddin S.H., Mustafa F.H., Alfouzan W.A., Alissa M., Alshengeti A., Almaghrabi R.H. (2023). Current and Future Technologies for the Detection of Antibiotic-Resistant Bacteria. Diagnostics.

[9] Máiz Carro L., Martínez-García M.A. (2019). Nebulized Hypertonic Saline in Noncystic Fibrosis Bronchiectasis: A Comprehensive Review. Ther. Adv. Respir. Dis..

[10] Shapiro D.J., Hall M., Neuman M.I., Hersh A.L., Cotter J.M., Cogen J.D., Brogan T.V., Ambroggio L., Blaschke A.J., Lipsett S.C. (2024). Outpatient Antibiotic Use and Treatment Failure Among Children With Pneumonia. JAMA Netw. Open.

[11] Bradley J.S., Byington C.L., Shah S.S., Alverson B., Carter E.R., Harrison C., Kaplan S.L., Mace S.E., McCracken G.H., Moore M.R. (2011). The Management of Community-Acquired Pneumonia in Infants and Children Older than 3 Months of Age: Clinical Practice Guidelines by the Pediatric Infectious Diseases Society and the Infectious Diseases Society of America. Clin. Infect. Dis..

[12] Li C.-X., Zhang L., Yan Y.-R., Ding Y.-J., Lin Y.-N., Zhou J.-P., Li N., Li H.-P., Li S.-Q., Sun X.-W. (2021). A Narrative Review of Exploring Potential Salivary Biomarkers in Respiratory Diseases: Still on Its Way. J. Thorac. Dis..

[13] Dawson M., Wirtz D., Hanes J. (2003). Enhanced Viscoelasticity of Human Cystic Fibrotic Sputum Correlates with Increasing Microheterogeneity in Particle Transport*. J. Biol. Chem..

[14] Abrami M., Biasin A., Tescione F., Tierno D., Dapas B., Carbone A., Grassi G., Conese M., Di Gioia S., Larobina D. (2024). Mucus Structure, Viscoelastic Properties, and Composition in Chronic Respiratory Diseases. Int. J. Mol. Sci..

[15] Wu C.L., Yang D.I., Wang N.Y., Kuo H.T., Chen P.Z. (2002). Quantitative Culture of Endotracheal Aspirates in the Diagnosis of Ventilator-Associated Pneumonia in Patients with Treatment Failure. Chest.

[16] Butler C.C., Kelly M.J., Hood K., Schaberg T., Melbye H., Serra-Prat M., Blasi F., Little P., Verheij T., Mölstad S. (2011). Antibiotic Prescribing for Discoloured Sputum in Acute Cough/Lower Respiratory Tract Infection. Eur. Respir. J..

[17] Zaragoza R., Vidal-Cortés P., Aguilar G., Borges M., Diaz E., Ferrer R., Maseda E., Nieto M., Nuvials F.X., Ramirez P. (2020). Update of the Treatment of Nosocomial Pneumonia in the ICU. Crit. Care.

[18] Woodhead M., Blasi F., Ewig S., Garau J., Huchon G., Ieven M., Ortqvist A., Schaberg T., Torres A., van der Heijden G. (2011). Guidelines for the Management of Adult Lower Respiratory Tract Infections--Full Version. Clin. Microbiol. Infect..

[19] Mandell L.A., Wunderink R.G., Anzueto A., Bartlett J.G., Campbell G.D., Dean N.C., Dowell S.F., File T.M., Musher D.M., Niederman M.S. (2007). Infectious Diseases Society of America/American Thoracic Society Consensus Guidelines on the Management of Community-Acquired Pneumonia in Adults. Clin. Infect. Dis..

[20] Bloise-Sánchez I., Alonso-Acero, L. en Nombre de la Comisión de Residentes y Jóvenes Especialistas de SEIMC (CoREIMC) (2024). Considerations on the Implementation of the Clinical Microbiology and Parasitology Training Program in Spanish Hospitals: A National Survey. Enfermedades Infecc. Microbiol. Clín. (Engl. Ed.).

[21] Azoulay E., Mokart D., Kouatchet A., Demoule A., Lemiale V. (2019). Acute Respiratory Failure in Immunocompromised Adults. Lancet Respir. Med..

[22] Chen Y.-S., Liao T.-Y., Hsu T.-C., Hsu W.-T., Lee M.-T.G., Lee C.-C. (2020). National Taiwan University Hospital Biomedical Data Science Research Group Temporal Trend and Survival Impact of Infection Source among Patients with Sepsis: A Nationwide Study. Crit. Care Resusc..

[23] Salam M.A., Al-Amin M.Y., Salam M.T., Pawar J.S., Akhter N., Rabaan A.A., Alqumber M.A.A. (2023). Antimicrobial Resistance: A Growing Serious Threat for Global Public Health. Healthcare.

[24] Jamshidi N., Waine M., Binet M., Mohan V., Carter D.J., Morgan B. (2024). The Adoption of Point of Care Testing Technologies for Respiratory Tract Infections in Primary Care in Australia: Challenges and Facilitators. Diagn. Microbiol. Infect. Dis..

[25] Stojanovic Z., Gonçalves-Carvalho F., Marín A., Abad Capa J., Domínguez J., Latorre I., Lacoma A., Prat-Aymerich C. (2022). Advances in Diagnostic Tools for Respiratory Tract Infections: From Tuberculosis to COVID-19—Changing Paradigms?. ERJ Open Res..

[26] Dheda K., Makambwa E., Esmail A. (2020). The Great Masquerader: Tuberculosis Presenting as Community-Acquired Pneumonia. Semin. Respir. Crit. Care Med..

[27] Frigati L., Greybe L., Andronikou S., Eber E., Venkatakrishna S.S.B., Goussard P. (2024). Respiratory Infections in Low and Middle-Income Countries. Paediatr. Respir. Rev..

[28] European Centre for Disease Prevention and Control Antimicrobial Resistance Surveillance in Europe 2023–2021 Data. https://www.ecdc.europa.eu/en/publications-data/antimicrobial-resistance-surveillance-europe-2023-2021-data.

[29] World Health Organization (WHO) Antimicrobial Resistance. https://www.who.int/news-room/fact-sheets/detail/antimicrobial-resistance.

[30] Enne V.I., Personne Y., Grgic L., Gant V., Zumla A. (2014). Aetiology of Hospital-Acquired Pneumonia and Trends in Antimicrobial Resistance. Curr. Opin. Pulm. Med..

[31] Torres A., Cilloniz C., Niederman M.S., Menéndez R., Chalmers J.D., Wunderink R.G., van der Poll T. (2021). Pneumonia. Nat. Rev. Dis. Primers.

[32] American Thoracic Society, Infectious Diseases Society of America (2005). Guidelines for the Management of Adults with Hospital-Acquired, Ventilator-Associated, and Healthcare-Associated Pneumonia. Am. J. Respir. Crit. Care Med..

[33] Eshwara V.K., Mukhopadhyay C., Rello J. (2020). Community-Acquired Bacterial Pneumonia in Adults: An Update. Indian. J. Med. Res..

[34] Allen K.D. (1991). Penicillin-Resistant Pneumococci. J. Hosp. Infect..

[35] Mameli C., Zuccotti G.V. (2013). The Impact of Viral Infections in Children with Community-Acquired Pneumonia. Curr. Infect. Dis. Rep..

[36] Ito A., Ishida T. (2020). Diagnostic Markers for Community-Acquired Pneumonia. Ann. Transl. Med..

[37] Falcone M., Venditti M., Shindo Y., Kollef M.H. (2011). Healthcare-Associated Pneumonia: Diagnostic Criteria and Distinction from Community-Acquired Pneumonia. Int. J. Infect. Dis..

[38] Dela Cruz C.S., Evans S.E., Restrepo M.I., Dean N., Torres A., Amara-Elori I., Awasthi S., Caler E., Cao B., Chalmers J.D. (2021). Understanding the Host in the Management of Pneumonia. An Official American Thoracic Society Workshop Report. Ann. Am. Thorac. Soc..

[39] Pasikhova Y., Ludlow S., Baluch A. (2017). Fever in Patients with Cancer. Cancer Control.

[40] Bouza E., Brun-Buisson C., Chastre J., Ewig S., Fagon J.-Y., Marquette C.H., Muñoz P., Niederman M.S., Papazian L., Rello J. (2001). Ventilator-Associated Pneumonia: European Task Force on Ventilator-Associated Pneumonia Chairmen of the Task Force: A. Torres and J. Carlet. Eur. Respir. J..

[41] Li Y., Roberts J.A., Walker M.M., Aslan A.T., Harris P.N.A., Sime F.B. (2024). The Global Epidemiology of Ventilator-Associated Pneumonia Caused by Multi-Drug Resistant *Pseudomonas aeruginosa*: A Systematic Review and Meta-Analysis. Int. J. Infect. Dis..

[42] Wasserman M.G., Graham R.J., Mansbach J.M. (2022). Airway Bacterial Colonization, Biofilms and Blooms, and Acute Respiratory Infection. Pediatr. Crit. Care Med..

[43] Alnimr A. (2023). Antimicrobial Resistance in Ventilator-Associated Pneumonia: Predictive Microbiology and Evidence-Based Therapy. Infect. Dis. Ther..

[44] Blot S., Ruppé E., Harbarth S., Asehnoune K., Poulakou G., Luyt C.-E., Rello J., Klompas M., Depuydt P., Eckmann C. (2022). Healthcare-Associated Infections in Adult Intensive Care Unit Patients: Changes in Epidemiology, Diagnosis, Prevention and Contributions of New Technologies. Intensive Crit. Care Nurs..

[45] Kim V., Criner G.J. (2013). Chronic Bronchitis and Chronic Obstructive Pulmonary Disease. Am. J. Respir. Crit. Care Med..

[46] Boers E., Barrett M., Su J.G., Benjafield A.V., Sinha S., Kaye L., Zar H.J., Vuong V., Tellez D., Gondalia R. (2023). Global Burden of Chronic Obstructive Pulmonary Disease Through 2050. JAMA Netw. Open.

[47] Nigro M., Laska I.F., Traversi L., Simonetta E., Polverino E. (2024). Epidemiology of Bronchiectasis. Eur. Respir. Rev..

[48] Angelis A.D., Johnson E.D., Sutharsan S., Aliberti S. (2024). Exacerbations of Bronchiectasis. Eur. Respir. Rev..

[49] Hess D.R. (2023). Respiratory Care Management of COPD Exacerbations. Respir. Care.

[50] Vidaillac C., Chotirmall S.H. (2021). *Pseudomonas aeruginosa* in Bronchiectasis: Infection, Inflammation, and Therapies. Expert Rev. Respir. Med..

[51] Oliver A., Cantón R., Campo P., Baquero F., Blázquez J. (2000). High Frequency of Hypermutable *Pseudomonas aeruginosa* in Cystic Fibrosis Lung Infection. Science.

[52] Thi M.T.T., Wibowo D., Rehm B.H.A. (2020). *Pseudomonas aeruginosa* Biofilms. Int. J. Mol. Sci..

[53] Martínez-García M.Á., Faner R., Oscullo G., la Rosa-Carrillo D., Soler-Cataluña J.J., Ballester M., Muriel A., Agusti A. (2022). Chronic Bronchial Infection Is Associated with More Rapid Lung Function Decline in Chronic Obstructive Pulmonary Disease. Ann. Am. Thorac. Soc..

[54] Cosío B.G., Shafiek H., Verdú J., Fiorentino F., Valera J.L., Martínez R., Romero S., Ramón L., Toledo-Pons N., Sala E. (2021). Implementation of an Integrated Care Model for Frequent-Exacerbator COPD Patients: A Controlled Prospective Study. Arch. Bronconeumol..

[55] Park H.-J., Woo A., Cha J.M., Lee K.-S., Lee M.-Y. (2018). Closed-Type Pre-Treatment Device for Point-of-Care Testing of Sputum. Sci. Rep..

[56] George S., Xu Y., Rodger G., Morgan M., Sanderson N.D., Hoosdally S.J., Thulborn S., Robinson E., Rathod P., Walker A.S. (2020). DNA Thermo-Protection Facilitates Whole-Genome Sequencing of Mycobacteria Direct from Clinical Samples. J. Clin. Microbiol..

[57] Saraswathy Veena V., Sara George P., Jayasree K., Sujathan K. (2015). Comparative Analysis of Cell Morphology in Sputum Samples Homogenized with Dithiothreitol, N-Acetyl-L Cysteine, Cytorich^®^ Red Preservative and in Cellblock Preparations to Enhance the Sensitivity of Sputum Cytology for the Diagnosis of Lung Cancer. Diagn. Cytopathol..

[58] Terranova L., Oriano M., Teri A., Ruggiero L., Tafuro C., Marchisio P., Gramegna A., Contarini M., Franceschi E., Sottotetti S. (2018). How to Process Sputum Samples and Extract Bacterial DNA for Microbiota Analysis. Int. J. Mol. Sci..

[59] Mulvanny A., Pattwell C., Beech A., Southworth T., Singh D. (2022). Validation of Sputum Biomarker Immunoassays and Cytokine Expression Profiles in COPD. Biomedicines.

[60] Clemente A., Alba-Patiño A., Santopolo G., Rojo-Molinero E., Oliver A., Borges M., Aranda M., del Castillo A., de la Rica R. (2021). Immunodetection of Lung IgG and IgM Antibodies against SARS-CoV-2 via Enzymatic Liquefaction of Respiratory Samples from COVID-19 Patients. Anal. Chem..

[61] Dippenaar A., Ismail N., Grobbelaar M., Oostvogels S., de Vos M., Streicher E.M., Heupink T.H., van Rie A., Warren R.M. (2022). Optimizing Liquefaction and Decontamination of Sputum for DNA Extraction from *Mycobacterium tuberculosis*. Tuberculosis.

[62] Clemente A., Alba-Patiño A., Rojo-Molinero E., Russell S.M., Borges M., Oliver A., de la Rica R. (2020). Rapid Detection of *Pseudomonas aeruginosa* Biofilms via Enzymatic Liquefaction of Respiratory Samples. ACS Sens..

[63] Santopolo G., Clemente A., Rojo-Molinero E., Fernández S., Álvarez M.C., Oliver A., de la Rica R. (2022). Improved Cytometric Analysis of Untouched Lung Leukocytes by Enzymatic Liquefaction of Sputum Samples. Biol. Proced. Online.

[64] Cartuliares M.B., Skjøt-Arkil H., Mogensen C.B., Skovsted T.A., Andersen S.L., Pedersen A.K., Rosenvinge F.S. (2023). Gram Stain and Culture of Sputum Samples Detect Only Few Pathogens in Community-Acquired Lower Respiratory Tract Infections: Secondary Analysis of a Randomized Controlled Trial. Diagnostics.

[65] Pouly O., Lecailtel S., Six S., Préau S., Wallet F., Nseir S., Rouzé A. (2020). Accuracy of Ventilator-Associated Events for the Diagnosis of Ventilator-Associated Lower Respiratory Tract Infections. Ann. Intensive Care.

[66] Ioanas M., Ferrer R., Angrill J., Ferrer M., Torres A. (2001). Microbial Investigation in Ventilator-Associated Pneumonia. Eur. Respir. J..

[67] Torres A., Fàbregas N., Ewig S., de la Bellacasa J.P., Bauer T.T., Ramirez J. (2000). Sampling Methods for Ventilator-Associated Pneumonia: Validation Using Different Histologic and Microbiological References. Crit. Care Med..

[68] Enne V.I., Aydin A., Baldan R., Owen D.R., Richardson H., Ricciardi F., Russell C., Nomamiukor-Ikeji B.O., Swart A.-M., High J. (2022). Multicentre Evaluation of Two Multiplex PCR Platforms for the Rapid Microbiological Investigation of Nosocomial Pneumonia in UK ICUs: The INHALE WP1 Study. Thorax.

[69] El-Nawawy A.A., Antonios M.A., Tawfik M.E., Meheissen M.A. (2022). Comparison of a Point-of-Care FilmArray Test to Standard-of-Care Microbiology Test in Diagnosis of Healthcare Associated Infections in a Tertiary Care Pediatric Intensive Care Unit. Antibiotics.

[70] Poole S., Tanner A.R., Naidu V.V., Borca F., Phan H., Saeed K., Grocott M.P.W., Dushianthan A., Moyses H., Clark T.W. (2022). Molecular Point-of-Care Testing for Lower Respiratory Tract Pathogens Improves Safe Antibiotic de-Escalation in Patients with Pneumonia in the ICU: Results of a Randomised Controlled Trial. J. Infect..

[71] Cojuc-Konigsberg G., Moscona-Nissan A., Guijosa A., Mireles Dávalos C.D., Martínez M.E.J., Mújica Sánchez M.A., Hernández Huizar V.F., Durán Barrón M.A., Gómez K.V., Andrade-Galindo R. (2023). Diagnostic Accuracy of the BioFire^®^ FilmArray^®^ Pneumonia Panel in COVID-19 Patients with Ventilator-Associated Pneumonia. BMC Infect. Dis..

[72] Søgaard K.K., Hinic V., Goldenberger D., Gensch A., Schweitzer M., Bättig V., Siegemund M., Bassetti S., Bingisser R., Tamm M. (2024). Evaluation of the Clinical Relevance of the Biofire© FilmArray Pneumonia Panel among Hospitalized Patients. Infection.

[73] Yoo I.Y., Huh K., Shim H.J., Yun S.A., Chung Y.N., Kang O.K., Huh H.J., Lee N.Y. (2020). Evaluation of the BioFire FilmArray Pneumonia Panel for Rapid Detection of Respiratory Bacterial Pathogens and Antibiotic Resistance Genes in Sputum and Endotracheal Aspirate Specimens. Int. J. Infect. Dis..

[74] Ince B., Sezgintürk M.K. (2022). Lateral Flow Assays for Viruses Diagnosis: Up-to-Date Technology and Future Prospects. Trends Anal. Chem..

[75] Shoemark A., Cant E., Carreto L., Smith A., Oriano M., Keir H.R., Perea L., Canto E., Terranova L., Vidal S. (2019). A Point-of-Care Neutrophil Elastase Activity Assay Identifies Bronchiectasis Severity, Airway Infection and Risk of Exacerbation. Eur. Respir. J..

[76] Wong A.Y.W., Johnsson A.T.A., Ininbergs K., Athlin S., Özenci V. (2021). Comparison of Four Streptococcus Pneumoniae Urinary Antigen Tests Using Automated Readers. Microorganisms.

[77] Cohen J.F., Bertille N., Cohen R., Chalumeau M. (2016). Rapid Antigen Detection Test for Group A Streptococcus in Children with Pharyngitis. Cochrane Database Syst. Rev..

[78] Shimada T., Noguchi Y., Jackson J.L., Miyashita J., Hayashino Y., Kamiya T., Yamazaki S., Matsumura T., Fukuhara S. (2009). Systematic Review and Metaanalysis: Urinary Antigen Tests for Legionellosis. Chest.

[79] Omidfar K., Riahi F., Kashanian S. (2023). Lateral Flow Assay: A Summary of Recent Progress for Improving Assay Performance. Biosensors.

[80] Yzerman E.P.F., Boer J.W.d., Lettinga K.D., Schellekens J., Dankert J., Peeters M. (2002). Sensitivity of Three Urinary Antigen Tests Associated with Clinical Severity in a Large Outbreak of Legionnaires’ Disease in The Netherlands. J. Clin. Microbiol..

[81] Dinh A., Duran C., Davido B., Lagrange A., Sivadon-Tardy V., Bouchand F., Beauchet A., Gaillard J.-L., Beaune S., Salomon J. (2018). Cost Effectiveness of Pneumococcal Urinary Antigen in Emergency Department: A Pragmatic Real-Life Study. Intern. Emerg. Med..

[82] Jenkins S., Ledeboer N.A., Westblade L.F., Burnham C.-A.D., Faron M.L., Bergman Y., Yee R., Mesich B., Gerstbrein D., Wallace M.A. (2020). Evaluation of NG-Test Carba 5 for Rapid Phenotypic Detection and Differentiation of Five Common Carbapenemase Families: Results of a Multicenter Clinical Evaluation. J. Clin. Microbiol..

[83] Sundell N., Andersson L.-M., Brittain-Long R., Sundvall P.-D., Alsiö Å., Lindh M., Gustavsson L., Westin J. (2019). PCR Detection of Respiratory Pathogens in Asymptomatic and Symptomatic Adults. J. Clin. Microbiol..

[84] Anjum M.F., Zankari E., Hasman H. (2017). Molecular Methods for Detection of Antimicrobial Resistance. Microbiol. Spectr..

[85] Li L., Wang Q., Zhang H., Yang M., Khan M.I., Zhou X. (2016). Sensor Histidine Kinase Is a β-Lactam Receptor and Induces Resistance to β-Lactam Antibiotics. Proc. Natl. Acad. Sci. USA.

[86] Kundu S., Varshney R., Sulabh S. (2024). Exploration of Isothermal Nucleic Acid Amplification Techniques in the Biomedical Field. Gene Genome Ed..

[87] Zhao Y., Chen F., Li Q., Wang L., Fan C. (2015). Isothermal Amplification of Nucleic Acids. Chem. Rev..

[88] Oliveira B.B., Veigas B., Baptista P.V. (2021). Isothermal Amplification of Nucleic Acids: The Race for the Next “Gold Standard”. Front. Sens..

[89] Cao Q., Liang S., Lin F., Cao J., Wang L., Li H., Liu M., Wang Y., Zhao L., Cao X. (2022). Detection of Haemophilus Influenzae by Loop-Mediated Isothermal Amplification Coupled with Nanoparticle-Based Lateral Flow Biosensor Assay. BMC Microbiol..

[90] Wang Y., Zhao X., Zhou Y., Lu J., Yu H., Li S. (2022). Establishment and Application of Loop-Mediated Isothermal Amplification Coupled with Nanoparticle-Based Lateral Flow Biosensor (LAMP-LFB) for Visual and Rapid Diagnosis of Candida Albicans in Clinical Samples. Front. Bioeng. Biotechnol..

[91] Zhao F., Niu L., Nong J., Wang C., Wang J., Liu Y., Gao N., Zhu X., Wu L., Hu S. (2018). Rapid and Sensitive Detection of *Pseudomonas aeruginosa* Using Multiple Cross Displacement Amplification and Gold Nanoparticle-Based Lateral Flow Biosensor Visualization. FEMS Microbiol. Lett..

[92] Niu L., Zhao F., Chen J., Nong J., Wang C., Wang J., Gao N., Zhu X., Wu L., Hu S. (2018). Isothermal Amplification and Rapid Detection of Klebsiella Pneumoniae Based on the Multiple Cross Displacement Amplification (MCDA) and Gold Nanoparticle Lateral Flow Biosensor (LFB). PLoS ONE.

[93] Yang X., Chen X., Huang J., Chen Y., Zheng W., Chen W., Chen H., Lei S., Li S. (2023). Ultrafast, One-Step, Label-Based Biosensor Diagnosis Platform for the Detection of *Mycobacterium tuberculosis* in Clinical Applications. ACS Infect. Dis..

[94] Barreda-García S., González-Álvarez M.J., de-los-Santos-Álvarez N., Palacios-Gutiérrez J.J., Miranda-Ordieres A.J., Lobo-Castañón M.J. (2015). Attomolar Quantitation of *Mycobacterium tuberculosis* by Asymmetric Helicase-Dependent Isothermal DNA-Amplification and Electrochemical Detection. Biosens. Bioelectron..

[95] Hu S., Niu L., Zhao F., Yan L., Nong J., Wang C., Gao N., Zhu X., Wu L., Bo T. (2019). Identification of Acinetobacter Baumannii and Its Carbapenem-Resistant Gene blaOXA-23-like by Multiple Cross Displacement Amplification Combined with Lateral Flow Biosensor. Sci. Rep..

[96] Ryall B., Carrara M., Zlosnik J.E.A., Behrends V., Lee X., Wong Z., Lougheed K.E., Williams H.D. (2014). The Mucoid Switch in *Pseudomonas aeruginosa* Represses Quorum Sensing Systems and Leads to Complex Changes to Stationary Phase Virulence Factor Regulation. PLoS ONE.

[97] Peláez E.C., Estevez M.C., Mongui A., Menéndez M.-C., Toro C., Herrera-Sandoval O.L., Robledo J., García M.J., Portillo P.D., Lechuga L.M. (2020). Detection and Quantification of HspX Antigen in Sputum Samples Using Plasmonic Biosensing: Toward a Real Point-of-Care (POC) for Tuberculosis Diagnosis. ACS Infect. Dis..

[98] Vaquer A., Adrover-Jaume C., Clemente A., Viana J., Rodríguez R., Rojo-Molinero E., Oliver A., de la Rica R. (2024). OriPlex: Origami-Enabled Multiplexed Detection of Respiratory Pathogens. Biosens. Bioelectron..

[99] Gunasekaran D., Rostovsky I., Taussig D., Bar-Am T., Wine Y., Sal-Man N., Vernick S. (2024). A Dual-Channel Electrochemical Biosensor Enables Concurrent Detection of Pathogens and Antibiotic Resistance. Biosens. Bioelectron..

[100] Defoirdt T. (2018). Quorum-Sensing Systems as Targets for Antivirulence Therapy. Trends Microbiol..

[101] Chen X., Wang C., Zheng Q.Y., Hu W.-C., Xia X.-H. (2024). Emerging Advances in Biosensor Technologies for Quorum Sensing Signal Molecules. Anal. Bioanal. Chem..

[102] Díaz-Pérez S.P., Solis C.S., López-Bucio J.S., Valdez Alarcón J.J., Villegas J., Reyes-De la Cruz H., Campos-Garcia J. (2023). Pathogenesis in *Pseudomonas aeruginosa* PAO1 Biofilm-Associated Is Dependent on the Pyoverdine and Pyocyanin Siderophores by Quorum Sensing Modulation. Microb. Ecol..

[103] Rodriguez-Urretavizcaya B., Pascual N., Pastells C., Martin-Gomez M.T., Vilaplana L., Marco M.-P. (2021). Diagnosis and Stratification of *Pseudomonas aeruginosa* Infected Patients by Immunochemical Quantitative Determination of Pyocyanin From Clinical Bacterial Isolates. Front. Cell Infect. Microbiol..

[104] Pastells C., Pascual N., Sanchez-Baeza F., Marco M.-P. (2016). Immunochemical Determination of Pyocyanin and 1-Hydroxyphenazine as Potential Biomarkers of *Pseudomonas aeruginosa* Infections. Anal. Chem..

[105] Abdelaziz A.A., Kamer A.M.A., Al-Monofy K.B., Al-Madboly L.A. (2023). *Pseudomonas aeruginosa*’s Greenish-Blue Pigment Pyocyanin: Its Production and Biological Activities. Microb. Cell Fact..

[106] Adrover-Jaume C., Clemente A., Rodríguez-Urretavizcaya B., Vilaplana L., Marco M.P., Rojo-Molinero E., Oliver A., de la Rica R. (2023). A Paper Biosensor for Overcoming Matrix Effects Interfering with the Detection of Sputum Pyocyanin with Competitive Immunoassays. Microchim. Acta.

[107] Yue S.-J., Huang P., Wang W., Hu H.-B., Zhang X.-H. (2024). Development of a Whole-Cell Biosensor for Detection of Pyocyanin Based on a Transcriptional Regulation Factor. Sens. Actuators B Chem..

[108] Wen K.Y., Cameron L., Chappell J., Jensen K., Bell D.J., Kelwick R., Kopniczky M., Davies J.C., Filloux A., Freemont P.S. (2017). A Cell-Free Biosensor for Detecting Quorum Sensing Molecules in P. Aeruginosa-Infected Respiratory Samples. ACS Synth. Biol..

[109] Santopolo G., Rojo-Molinero E., Clemente A., Borges M., Oliver A., de la Rica R. (2021). Bedside Detection of Carbapenemase-Producing Pathogens with Plasmonic Nanosensors. Sens. Actuators B Chem..

[110] Jo H.J., Ryu J.S., Robby A.I., Kim Y.S., Chung H.J., Park S.Y. (2022). Rapid and Selective Electrochemical Sensing of Bacterial Pneumonia in Human Sputum Based on Conductive Polymer Dot Electrodes. Sens. Actuators B Chem..

[111] Fang W., Wu J., Cheng M., Zhu X., Du M., Chen C., Liao W., Zhi K., Pan W. (2023). Diagnosis of Invasive Fungal Infections: Challenges and Recent Developments. J. Biomed. Sci..

[112] Kumar S., Sharma R., Bhawna, Gupta A., Singh P., Kalia S., Thakur P., Kumar V. (2022). Prospects of Biosensors Based on Functionalized and Nanostructured Solitary Materials: Detection of Viral Infections and Other Risks. ACS Omega.

[113] Sivakumar R., Lee N.Y. (2022). Recent Advances in Airborne Pathogen Detection Using Optical and Electrochemical Biosensors. Anal. Chim. Acta.

[114] Tarim E.A., Karakuzu B., Oksuz C., Sarigil O., Kizilkaya M., Al-Ruweidi M.K.A.A., Yalcin H.C., Ozcivici E., Tekin H.C. (2021). Microfluidic-Based Virus Detection Methods for Respiratory Diseases. Emergent Mater..

[115] Gopal A., Yan L., Kashif S., Munshi T., Roy V.A.L., Voelcker N.H., Chen X. (2022). Biosensors and Point-of-Care Devices for Bacterial Detection: Rapid Diagnostics Informing Antibiotic Therapy. Adv. Healthc. Mater..

[116] Qureshi A., Niazi J.H. (2021). Biosensors for Detecting Viral and Bacterial Infections Using Host Biomarkers: A Review. Analyst.

